# Temperature-Dependent Electromechanical and Acoustic Responses of a Tubular PZT-5A Transducer: Finite-Element Modeling and Experimental Investigation

**DOI:** 10.3390/s26185947

**Published:** 2026-09-20

**Authors:** Ping Han, Baohai Tan, Kai Zhang, Yuanda Su, Zhicheng Zheng

**Affiliations:** 1 School of Geosciences and Technology, China University of Petroleum (East China), Qingdao 266555, China; z24010092@s.upc.edu.cn (P.H.); kai@upc.edu.cn (K.Z.); suyuanda@upc.edu.cn (Y.S.); z23010075@s.upc.edu.cn (Z.Z.); 2 State Key Laboratory of Deep Oil and Gas, China University of Petroleum (East China), Qingdao 266555, China

**Keywords:** PZT-5A, temperature-dependent finite-element model, impedance, laser vibrometry, acoustic radiation, acoustic logging

## Abstract

Temperature-induced variations in piezoelectric material properties can detune acoustic-logging transducers and alter their electrical, mechanical, and acoustic responses. This study establishes a temperature-dependent finite-element model of a tubular lead zirconate titanate (PZT-5A) transducer over 0–200 °C. Temperature-dependent dielectric, piezoelectric, and elastic parameters were implemented as constitutive functions. Frequency-domain impedance simulations were compared with impedance measurements over 20–200 °C, while transient fluid-loaded simulations and laser-vibrometry measurements were used to characterize the acoustic-pressure and vessel-wall vibration responses. The simulated resonant frequency decreased from 21.379 to 19.35 kHz, and the measured value decreased from 21.135 to 19.55 kHz. The simulated static capacitance increased from 18.8 to 81.0 nF, whereas the measured value increased from 23.7 to 93.0 nF. An isolated geometric-expansion sensitivity analysis indicated that omitted dimensional changes may partly account for this discrepancy. The fitted motional resistance decreased in both the simulations and experiments. The vessel-wall vibration-velocity squared integral decreased from 0.03483 to $0.0067{\mathrm{mm}}^{2}$/s, and the simulated acoustic-pressure squared integral decreased from 2.14926 to 1.59839 Pa^2^·s. The frequency corresponding to the maximum sound-pressure spectrum also decreased from 22.644 to 21.607 kHz. The agreement in the principal temperature-dependent trends indicates that the model captures the dominant electromechanical behavior, while the remaining capacitance discrepancy highlights the influence of thermal deformation and other nonideal experimental factors. The proposed framework provides a basis for performance prediction and temperature compensation of piezoelectric transducers used in acoustic logging and other elevated-temperature acoustic systems.

## 1. Introduction

Piezoelectric transducers are widely used in ultrasonic testing, nondestructive evaluation, acoustic sensing, actuation, and energy conversion because of their compact structure, rapid response, and efficient electromechanical conversion [1]. Their performance is especially critical in elevated-temperature environments, including power-generation equipment, nuclear systems, process monitoring, structural-health monitoring, and continuous in-service inspection [2,3,4,5,6,7]. High-temperature transducer development has therefore addressed not only active piezoelectric materials but also electrodes, bonding layers, backing materials, packaging, thermal cycling, and heat dissipation [8,9,10,11].

Deep subsurface exploration and acoustic logging impose similarly demanding thermal conditions. In acoustic-logging tools, piezoelectric transmitters generate monopole, dipole, or higher-order acoustic fields in a confined borehole-fluid environment. Their resonant frequency, impedance, vibration amplitude, and radiation response directly affect the excitation energy, received-signal quality, and reliability of formation evaluation [12,13]. With increasing downhole temperature, changes in dielectric, piezoelectric, and elastic properties can shift the operating frequency, alter the electrical load presented to the driving circuit, and reduce the acoustic output. Excitation and matching networks optimized at room temperature may therefore become suboptimal in service.

Lead zirconate titanate (PZT) ceramics remain widely used because they provide a practical balance among piezoelectric activity, electromechanical coupling, manufacturability, geometric flexibility, and cost [1]. Their constitutive properties, however, are temperature dependent. Measurements on PZT ceramics, films, and standard specimens have reported systematic changes in dielectric permittivity, dielectric loss, piezoelectric coefficients, elastic compliance, mechanical properties, and resonance spectra over broad temperature ranges [14,15,16,17,18,19,20,21,22,23]. Temperature-dependent field response and domain-wall depinning further show that these variations cannot be attributed solely to intrinsic lattice behavior [24]. Similar environmental sensitivity has also been observed at cryogenic temperatures and in piezoelectric sensing systems, confirming that the measured response depends on the coupled state of the material and device rather than on a single constant [25,26].

The macroscopic response of ferroelectric ceramics contains intrinsic lattice contributions and extrinsic contributions associated with domain-wall motion and domain switching. Classical weak-signal studies and direct diffraction measurements have demonstrated that domain processes contribute substantially to dielectric and piezoelectric behavior [27,28]. Numerical studies with realistic domain structures likewise show that domain configuration modifies the electromechanical response [29]. More recent alternating-current poling studies have shown that deliberately changing the domain configuration can strongly modify the dielectric permittivity and piezoelectric coefficients [30,31]. Although those studies address domain engineering rather than temperature directly, they support the interpretation that thermally activated changes in domain mobility can influence the effective constitutive properties of a transducer.

Previous studies have addressed the temperature dependence of piezoelectric systems at different levels. Material-level investigations have measured the temperature-dependent dielectric, piezoelectric, and elastic coefficients of PZT ceramics [15,16,18,19]. At the device level, Bilgunde and Bond quantified the effects of individual temperature-dependent material coefficients on the resonance modes of a PZT-5A disc [32], whereas Wellendorf et al. investigated the temperature-dependent resonance behavior of Langevin transducers using experimentally fitted finite-element models [33]. Bakaric et al. measured the temperature-dependent output of single-element PZT transducers using radiation-force and laser-vibrometry methods [34]. These studies have substantially advanced the understanding of temperature-dependent material properties, resonance behavior, or transducer output. However, these aspects have generally been investigated separately, and the relationships among the electrical, mechanical, and acoustic responses have not been sufficiently established within a consistent temperature-dependent framework, particularly for low-frequency tubular transducers used in acoustic-logging applications.

Accordingly, this study investigates a tubular PZT-5A transducer over 0–200 °C. The scientific contribution does not arise from the use of finite-element analysis or laser vibrometry alone, but from establishing a cross-domain framework that connects temperature-dependent PZT-5A constitutive properties with the electrical impedance, mechanical vibration response, and fluid-loaded acoustic-pressure response of the transducer. Frequency-domain impedance simulations are compared with impedance measurements, while transient acoustic-radiation simulations and laser-vibrometry measurements are used to examine the relationships among the electrical, mechanical, and acoustic response indicators. In addition, an electromagnetic-hammer control experiment is used to evaluate the temperature-dependent influence of the silicone-oil–vessel system independently of the transducer, and a separate geometric-expansion sensitivity analysis is performed to identify one possible source of the discrepancy between simulated and measured static capacitance. The objective is therefore to provide a physically interpretable connection between temperature-dependent material properties and multiple device-level responses, rather than only an engineering characterization of an individual performance parameter.

## 2. Materials and Methods

### 2.1. Tubular Piezoelectric Transducer and Its Parameters

The investigated device was a radially polarized PZT-5A tubular transducer with an outer diameter of 70 mm, an inner diameter of 60 mm, a height of 76 mm, and a wall thickness of 5 mm. Continuous electrodes covered the inner and outer cylindrical surfaces. The tubular geometry is representative of axisymmetric transmitters used in acoustic-logging and underwater-acoustic systems. PZT-5A was selected because of its relatively high piezoelectric activity and electromechanical coupling, while its Curie temperature is sufficiently above the investigated range of 0–200 °C [1,16].

### 2.2. Finite Element Simulation

#### 2.2.1. Establishment of the Geometric Model

**Figure 1:**
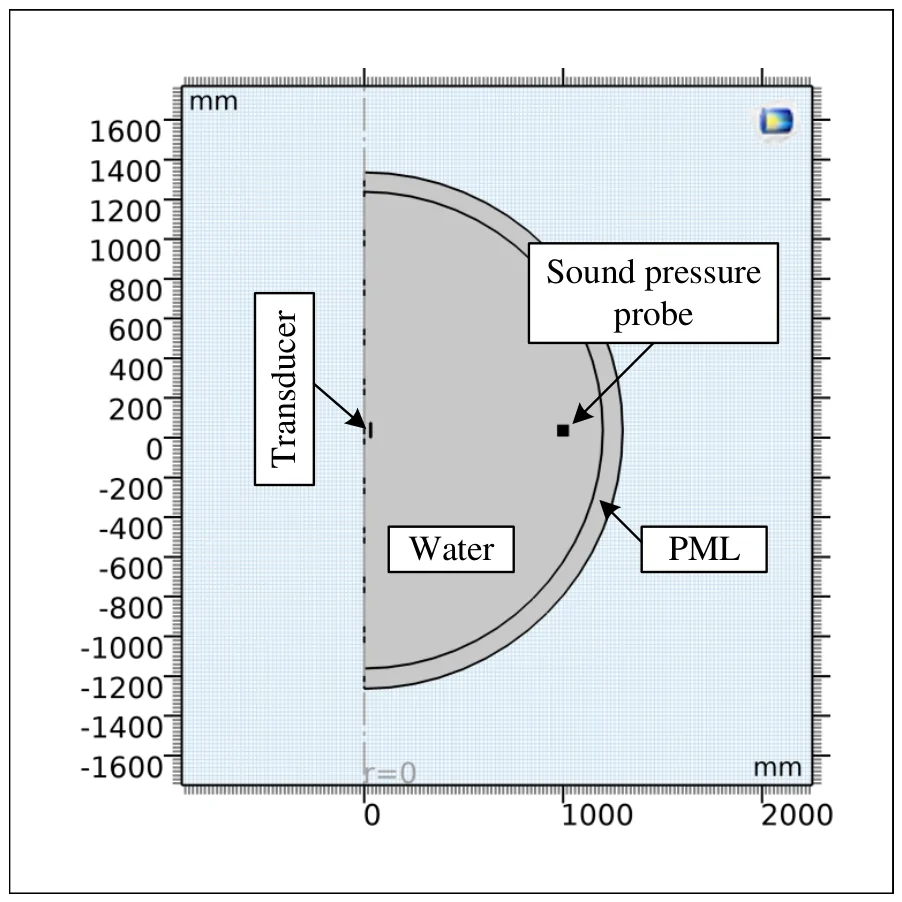
Axisymmetric FE model of the tubular transducer and surrounding spherical water domain.

COMSOL Multiphysics 6.1 was used to construct a two-dimensional axisymmetric FE model of the tubular transducer. For the fluid-loaded transient acoustic simulation, the transducer was surrounded by a spherical water domain with a radius of 1.2 m, as shown in Figure 1. A sound-pressure probe was placed 1 m radially from the transducer center. A perfectly matched layer (PML) with a thickness of 0.1 m was added outside the physical water domain to attenuate outgoing waves and reduce artificial reflections. For the impedance simulations, the water domain and PML were disabled so that only the electrically driven ceramic was retained.

#### 2.2.2. Construction of a PZT-5A Material Model Incorporating Temperature-Dependent Functions

The PZT-5A piezoelectric ceramic was modeled using the linear piezoelectric constitutive equations in the strain–charge form:

(1) $$S=s^{E}T+d^{T}E,$$

(2) $$D=dT+\varepsilon^{T}E,$$

where S and T denote the strain and stress vectors, respectively; E and D are the electric-field and electric-displacement vectors; $s^{E}$ is the elastic compliance matrix at constant electric field; d is the piezoelectric strain-coefficient matrix; $d^{T}$ denotes its transpose; and $\varepsilon^{T}$ is the dielectric-permittivity matrix at constant stress. The material 3-axis was aligned with the radial polarization direction of the tube. The bold stress vector T is distinct from the scalar temperature parameter Θ introduced below.

Because the polarized PZT-5A ceramic is transversely isotropic about the polarization direction, the elastic compliance matrix can be expressed as

(3) $$s^{E}=\left[\begin{array}{cccccc} s_{11}^{E} & s_{12}^{E} & s_{13}^{E} & 0 & 0 & 0\\ s_{12}^{E} & s_{11}^{E} & s_{13}^{E} & 0 & 0 & 0\\ s_{13}^{E} & s_{13}^{E} & s_{33}^{E} & 0 & 0 & 0\\ 0 & 0 & 0 & s_{55}^{E} & 0 & 0\\ 0 & 0 & 0 & 0 & s_{55}^{E} & 0\\ 0 & 0 & 0 & 0 & 0 & 2\left(s_{11}^{E}-s_{12}^{E}\right) \end{array}\right].$$

The dielectric-permittivity matrix is given by

(4) $$\varepsilon^{T}=\varepsilon_{0}\left[\begin{array}{ccc} \varepsilon_{11}^{T}/\varepsilon_{0} & 0 & 0\\ 0 & \varepsilon_{11}^{T}/\varepsilon_{0} & 0\\ 0 & 0 & \varepsilon_{33}^{T}/\varepsilon_{0} \end{array}\right],$$

where $\varepsilon_{0}$ is the vacuum permittivity.

The piezoelectric strain-coefficient matrix is expressed as

(5) $$d=\left[\begin{array}{cccccc} 0 & 0 & 0 & 0 & d_{15} & 0\\ 0 & 0 & 0 & d_{15} & 0 & 0\\ d_{31} & d_{31} & d_{33} & 0 & 0 & 0 \end{array}\right].$$

The temperature-dependent functions used for PZT-5A were constructed primarily from two experimental datasets. Hooker directly measured the dielectric and piezoelectric properties of commercial PZT-5A over a temperature range of −150 to 250 °C. The dielectric properties were measured using an LCR meter, whereas the transverse and longitudinal piezoelectric coefficients, $d_{31}$ and $d_{33}$, were determined from the resonance and antiresonance characteristics of standard piezoelectric specimens [16]. For the anisotropic dielectric and elastic parameters that were not completely reported for PZT-5A by Hooker, the temperature-dependent full-matrix measurements of soft EC-65 PZT reported by Sabat et al. were used [15]. EC-65 is a Navy Type-II soft PZT commonly regarded as an equivalent counterpart of PZT-5A. Sabat et al. determined the dielectric, piezoelectric, and elastic-compliance coefficients by analyzing the fundamental impedance or admittance resonances of five standard specimen geometries over −165 to 195 °C. Specifically, the temperature trends of $\varepsilon_{11}^{T}$, $\varepsilon_{33}^{T}$, $d_{31}$, $d_{33}$, $s_{11}^{E}$, and $s_{12}^{E}$ were obtained from these two datasets and normalized to the PZT-5A values at 20 °C provided by Baoding Hongsheng Acoustic Devices Co., Ltd. (Baoding, China).

Table 1 lists the temperature values used in the simulations and the corresponding values calculated from the temperature-dependent functions. The coefficients $d_{15}$, $s_{13}^{E}$, $s_{33}^{E}$, and $s_{55}^{E}$ were retained at their room-temperature values, whereas the parameters listed in Table 1 were defined as temperature-dependent functions.

#### 2.2.3. Simulation Objectives and Excitation Methods

Different excitation methods were employed for the two simulation objectives considered in this study. During the impedance simulation, the inner surface of the piezoelectric ceramic was grounded, and a voltage with an amplitude of 1 V was applied to the outer surface. The complex port-current response, I(ω), was then obtained from the frequency-domain simulation.

According to the definition of admittance, the port admittance is expressed as

(6) $$Y\left(\omega \right)=\frac{I(\omega )}{V_{0}},$$

where I(ω) is the complex port current and $V_{0}$ is the amplitude of the applied voltage. Accordingly, the port impedance is given by

(7) $$Z\left(\omega \right)=\frac{1}{Y(\omega )}=\frac{V_{0}}{I(\omega )}.$$

When $V_{0}=1\;V$, the port admittance is numerically equal to the complex port current:

(8) Y(ω)=I(ω).

**Figure 2:**
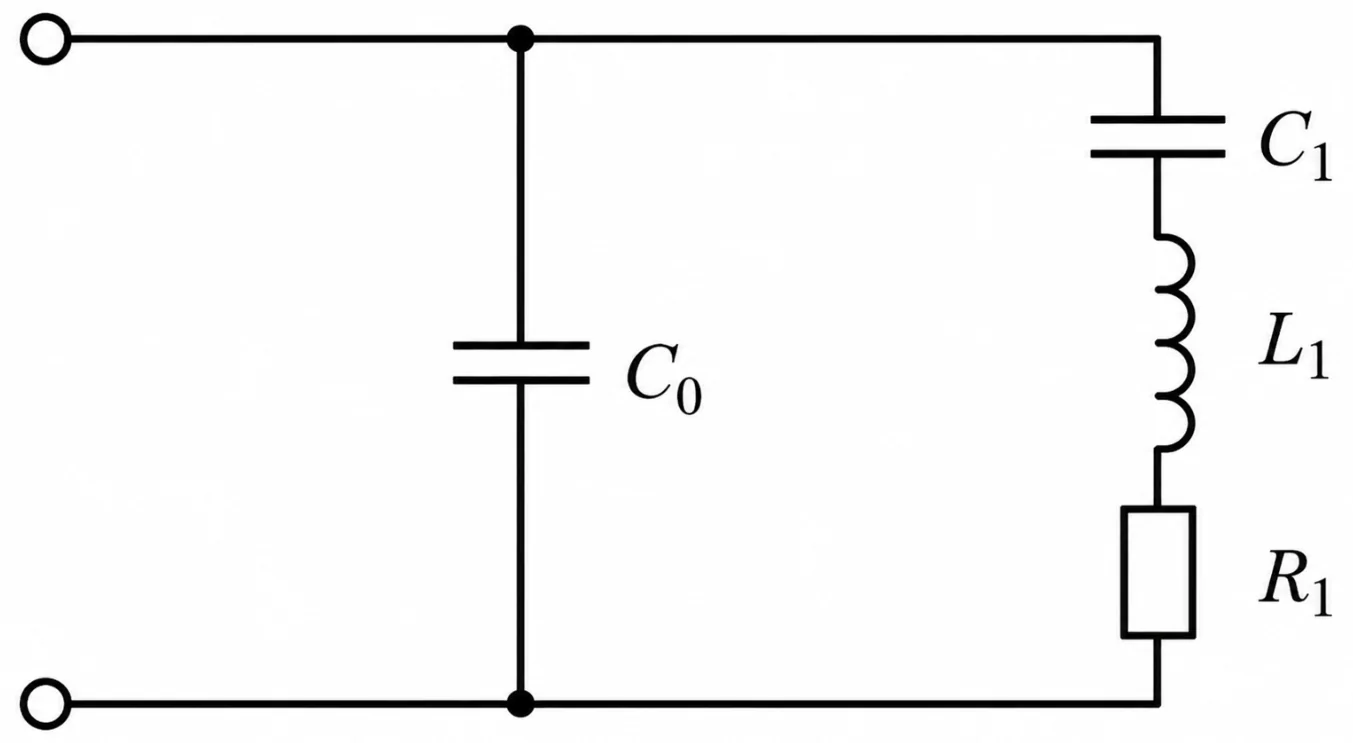
Butterworth–Van Dyke equivalent circuit used to represent the dominant resonance.

The complex port current is numerically equal to the admittance when $V_{0}=1\;V$. The Butterworth–Van Dyke (BVD) circuit in Figure 2 was used to represent the input electrical behavior near the dominant radial mode [35]. It consists of the static capacitance, $C_{0}$, in parallel with a motional branch containing $R_{1}$, $L_{1}$, and $C_{1}$. The same BVD representation was used when comparing the equivalent parameters obtained from the simulated and measured admittance responses.

During the transient acoustic-radiation simulation, the inner surface of the piezoelectric ceramic was grounded, and a three-cycle sinusoidal voltage signal with an amplitude of 1 V was applied to the outer surface. The excitation frequency was set to the resonant frequency of the piezoelectric ceramic obtained from the impedance simulation at room temperature (25 °C). This excitation method was used to analyze the acoustic-radiation characteristics of the transducer in water.

**Figure 3:**
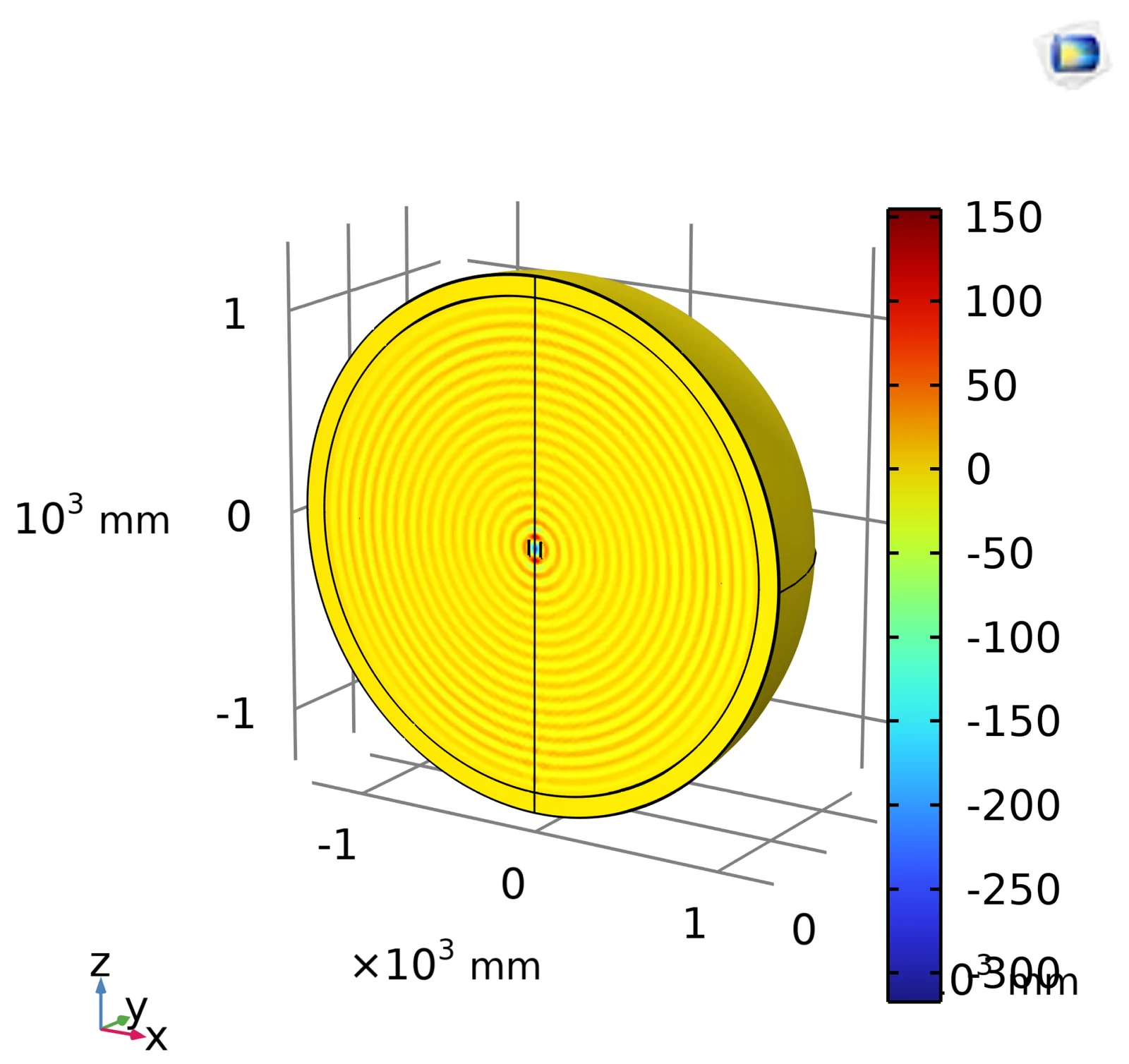
Cross-sectional view of the acoustic field in the spherical water domain at 800 μs.

The sound-pressure response was extracted using the sound-pressure probe positioned at a radial distance of 1 m from the center of the transducer. The recorded sound pressure was used to characterize the conversion capability of the piezoelectric transducer from electrical voltage input to acoustic-pressure output. Figure 3 shows a cross-sectional view of the simulated acoustic field in the two-dimensional axisymmetric spherical water model.

#### 2.2.4. Physics Interfaces and Boundary Conditions

The electromechanical response of the tubular PZT-5A transducer was modeled using the Solid Mechanics and Electrostatics physics interfaces, which were coupled through the Piezoelectric Effect multiphysics interface. The constitutive behavior of the piezoelectric ceramic was described by Equations (1) and (2), with the temperature-dependent material matrices defined in Section 2.2.2. No free volume charge or external volumetric mechanical force was prescribed within the piezoelectric domain.

For the impedance simulation, only the piezoelectric ceramic domain was retained, and a frequency-domain study was performed. The electrode on the inner cylindrical surface was assigned the ground boundary condition, whereas an electric potential of 1 V was applied to the outer electrode. The remaining nonelectrode surfaces were electrically insulated using the zero-charge condition. Except for the axisymmetric constraint, the mechanical surfaces of the ceramic were assigned the free boundary condition, allowing the transducer to vibrate freely under electrical excitation.

For the transient acoustic-radiation simulation, the pressure acoustics–transient interface was introduced to describe acoustic-wave propagation in the surrounding water domain. The acoustic–structure boundary multiphysics coupling was applied at the ceramic–water interface, through which the normal motion of the ceramic generated acoustic waves in the water and the acoustic pressure simultaneously acted as a mechanical load on the ceramic surface. A perfectly matched layer with a thickness of 0.1 m was placed outside the physical water domain to absorb outwardly propagating waves and minimize artificial reflections from the outer boundary.

#### 2.2.5. Mesh Configuration and Study Settings

To ensure the computational accuracy of the electromechanical coupling response inside the piezoelectric ceramic and acoustic-wave propagation in the surrounding water domain, a combination of physics-controlled meshing and local mesh refinement was adopted. Free triangular meshes were used in the piezoelectric ceramic and water domains. Local mesh refinement was applied within the piezoelectric ceramic, along the inner and outer electrode boundaries, and at the acoustic–structure coupling boundary between the ceramic and the water domain to accurately resolve the relatively large displacement, electric-field, and acoustic-pressure gradients near resonance. A mapped mesh arranged along the acoustic-wave propagation direction was employed in the PML to reduce numerical reflections caused by abrupt mesh transitions and to ensure the gradual attenuation of acoustic waves within the PML.

The maximum element size in the acoustic domain was determined according to the shortest wavelength corresponding to the highest analysis frequency:

(9) $$\lambda_{\min}=\frac{c_{w}}{f_{\max}},$$

where $c_{w}$ is the speed of sound in water and $f_{\max}$ is the highest frequency considered in the simulation. To ensure that each acoustic wavelength contained a sufficient number of mesh elements, the maximum element size in the water domain was constrained by

(10) $$h_{\max}\leq \frac{\lambda_{\min}}{6}.$$

For the piezoelectric ceramic domain, the mesh size was determined according to the shortest elastic wavelength within the material:

(11) $$\lambda_{\mathrm{PZT},\min}=\frac{c_{\mathrm{PZT},\min}}{f_{\max}},$$

where $c_{\mathrm{PZT},\min}$ is the minimum relevant elastic-wave velocity in PZT-5A for the vibration mode considered. Accordingly, the maximum element size in the piezoelectric ceramic domain was constrained by

(12) $$h_{\mathrm{PZT}}\leq \frac{\lambda_{\mathrm{PZT},\min}}{6}.$$

After the initial maximum element sizes had been determined, the mesh size was further reduced and the simulations were repeated. Mesh independence was considered to have been achieved when the relative variation in the monitored simulation result between two successive mesh refinements was less than 1%.

A parametric sweep was added to evaluate the temperature-dependent response of the transducer. The global temperature parameter Θ was varied from 273.15 to 473.15 K in increments of 20 K, corresponding to a temperature range of 0–200 °C. The temperature sweep was defined as

(13) $$\Theta =\mathrm{range}\left(\;273.15\;K,20\;K,473.15\;K\right).$$

For the impedance-characteristic simulation, a frequency-domain study was employed. The frequency range was set from 15 to 30 kHz, which completely covered the first radial resonance peak of the experimental transducer and the frequency-response regions on both sides of the resonance peak. The frequency increment was set to 0.001 kHz:

(14) $$f=\mathrm{range}\left(\;15\;\mathrm{kHz},0.001\;\mathrm{kHz},30\;\mathrm{kHz}\right).$$

For the transient acoustic-radiation simulation, a time-dependent study was employed. The simulation time ranged from 0 to 1500 μs, with a time increment of 1 μs:

(15) $$t=\mathrm{range}\left(\;0\;\mu s,1\;\mu s,1500\;\mu s\right).$$

This time interval completely covered the three-cycle excitation process, acoustic-wave propagation from the transducer to the sound-pressure probe located 1 m away, and the subsequent principal vibration and acoustic-pressure decay processes.

### 2.3. Impedance and Laser-Vibrometry Experiments

Although finite-element simulations can effectively predict variations in transducer performance at different temperatures, the simulation results are generally obtained under idealized conditions and cannot fully account for the complex factors present under actual operating conditions. Therefore, experimental assessment is required to evaluate the reliability of the simulation results and to further investigate the actual operating performance of the transducer under high-temperature conditions. To this end, impedance and laser-vibrometry experiments were conducted in this study, and the experimental results were compared with the corresponding simulation results.

**Figure 4:**
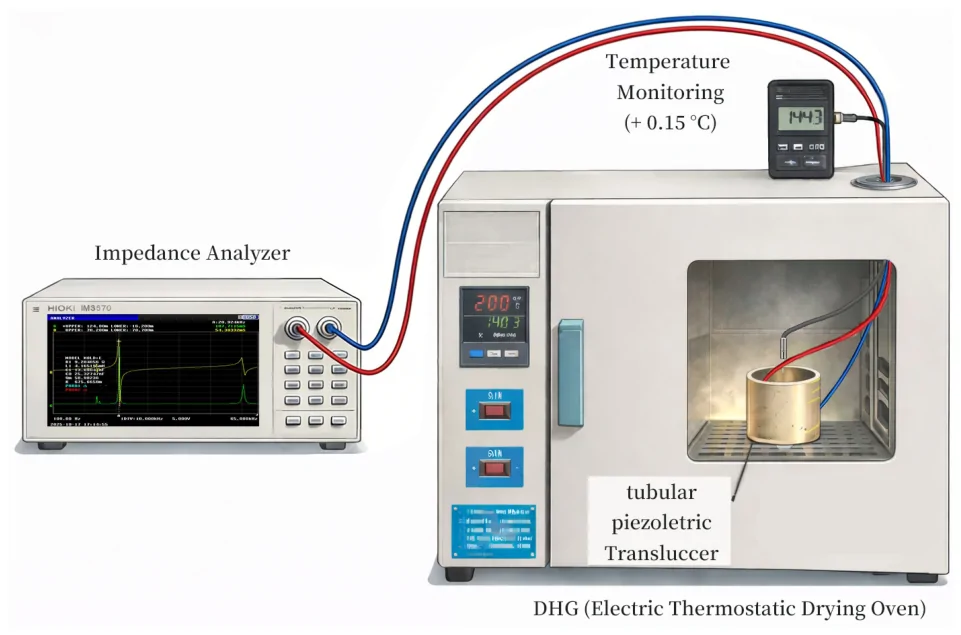
Diagram of the temperature-controlled impedance testing system.

In the impedance experiments, the transducer was heated in an electric constant-temperature drying oven (DHG) over a temperature range of 20–200 °C. The resonant frequency and equivalent-circuit parameters of the transducer at different temperatures were measured using a high-precision impedance analyzer (HIOKI IM3570). The experimental setup is shown in Figure 4. The temperature-control accuracy of the constant-temperature oven was ±0.2 °C. A temperature sensor with an accuracy of ±0.15 °C was placed near the transducer to monitor its temperature. At each temperature setpoint, the transducer was maintained under isothermal conditions for 10 min before measurement to promote thermal equilibration.

Although impedance testing can characterize the temperature-dependent variations in the electrical parameters and resonance characteristics of piezoelectric transducers, it essentially reflects the electrical response of the transducer. Variations in resonant frequency, static capacitance, and impedance spectra can only indirectly indicate the evolution of transducer performance and are insufficient to directly characterize its mechanical vibration output and excitation capability under actual operating conditions. Therefore, impedance experiments alone cannot provide a comprehensive evaluation of the temperature sensitivity of the transducer’s acoustic-radiation performance, and its mechanical excitation output must also be evaluated directly.

Conventional elevated-temperature acoustic testing generally employs a thermal shield between the transmitting and receiving transducers so that the transmitting transducer can be heated while the receiving transducer remains at a relatively stable temperature. However, it is difficult to simultaneously achieve effective thermal isolation and satisfactory acoustic transmission. Therefore, a high-temperature vessel filled with silicone oil was designed to seal and secure the transducer. The vibration of the outer wall of the high-temperature vessel was measured using a laser vibrometer to indirectly characterize the acoustic-radiation output of the piezoelectric transducer.

Because the acoustic waves generated by the transducer undergo multiple reflections and propagation attenuation inside the high-temperature vessel, it was necessary to evaluate the correlation between the vibration response of the vessel wall and the actual excitation output of the transducer. This comparison was used to assess whether vessel-wall vibration velocity could serve as a relative indicator of the transducer’s mechanical excitation output.

**Figure 5:**
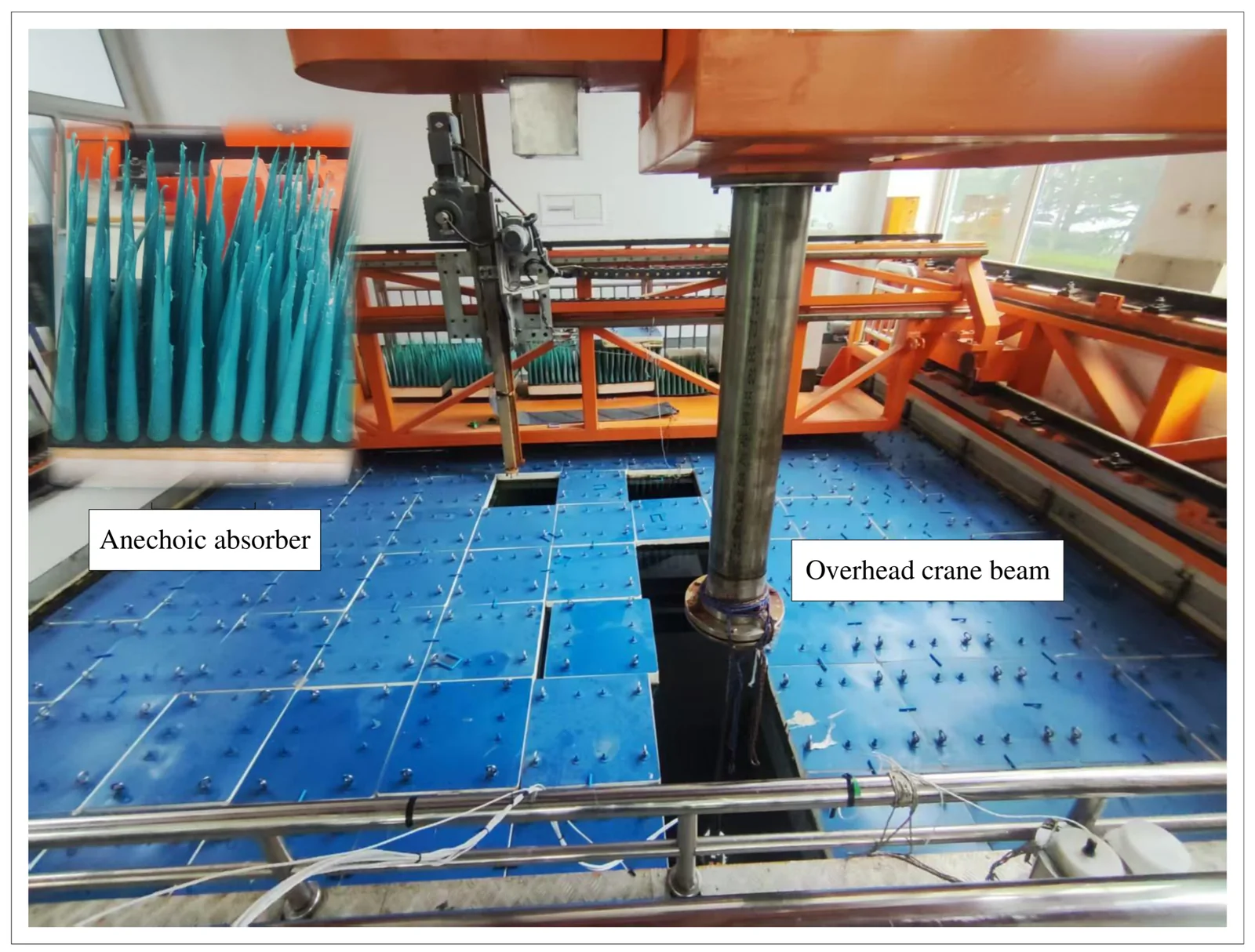
Anechoic water tank and the surrounding conical acoustic absorbers.

For this purpose, the same piezoelectric transducer was tested separately in an anechoic water tank and in the high-temperature vessel. In Group A, the transducer was placed in a large anechoic water tank, and the hydrophone-received voltage waveforms were recorded using an oscilloscope. In Group B, the vibration velocity of the outer wall of the high-temperature vessel was measured using a laser vibrometer. As shown in Figure 5, the anechoic water tank was cubic, with a side length of 6 m, and its inner boundaries were surrounded by conical acoustic absorbers. The piezoelectric transducer and hydrophone were suspended and fixed using an overhead support beam.

**Figure 6:**
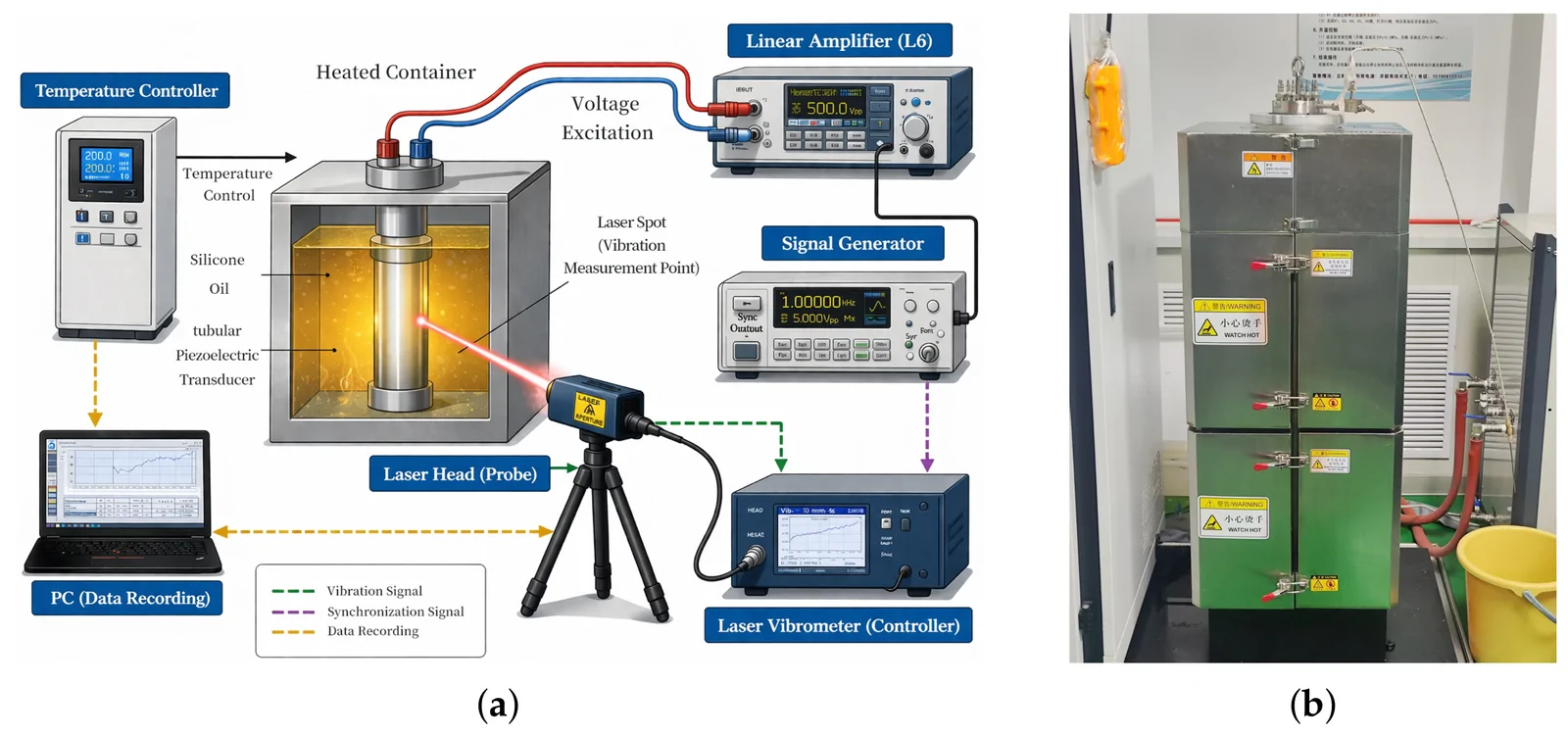
High-temperature vessel and laser-vibration measurement system: (**a**) schematic of the high-temperature vessel; (**b**) laser-vibration measurement setup.

Figure 6 shows the high-temperature vessel and the laser-vibrometry measurement setup. The experimental procedure was as follows:

1. A temperature controller was used to heat the sealed high-temperature vessel filled with silicone oil.
2. A signal generator produced a three-cycle low-voltage sinusoidal burst at a repetition rate of 3 Hz. The excitation frequency was set to the resonant frequency measured at 25 °C during the impedance experiment. The signal was amplified to the required high-voltage level by a linear power amplifier with a maximum output capability of 2000 V.
3. The amplified voltage signal was applied to the tubular piezoelectric transducer to generate acoustic waves. The acoustic waves propagated through the silicone oil and induced mechanical vibration of the outer wall of the high-temperature vessel.
4. The signal generator simultaneously provided a trigger signal to the laser vibrometer, enabling synchronized acquisition of the vessel-wall vibration response.

The custom high-temperature vessel had a temperature-control accuracy of ±0.2 °C. Conductive connectors were installed on the top of the vessel and connected to the electrodes on the inner and outer surfaces of the tubular transducer. A SOPTOP LV-SC400-P Doppler laser vibrometer with a sampling rate of 50 MHz was used to measure the vibration of the outer wall of the vessel at high temporal resolution. Before the experiments, the laser vibrometer was calibrated using a reference vibration source. Within the investigated range, the combined uncertainty associated with temperature control and vibration measurement was estimated from the instrument specifications and calibration to be approximately ±5%.

To examine the relationship between the acoustic response measured in the anechoic water tank and the vessel-wall vibration response measured by laser vibrometry, the excitation voltage applied to the piezoelectric transducer was increased from 200 to 1000 V in increments of 200 V. The hydrophone-received voltage waveforms and vessel-wall vibration-velocity waveforms were recorded at each excitation voltage.

**Figure 7:**
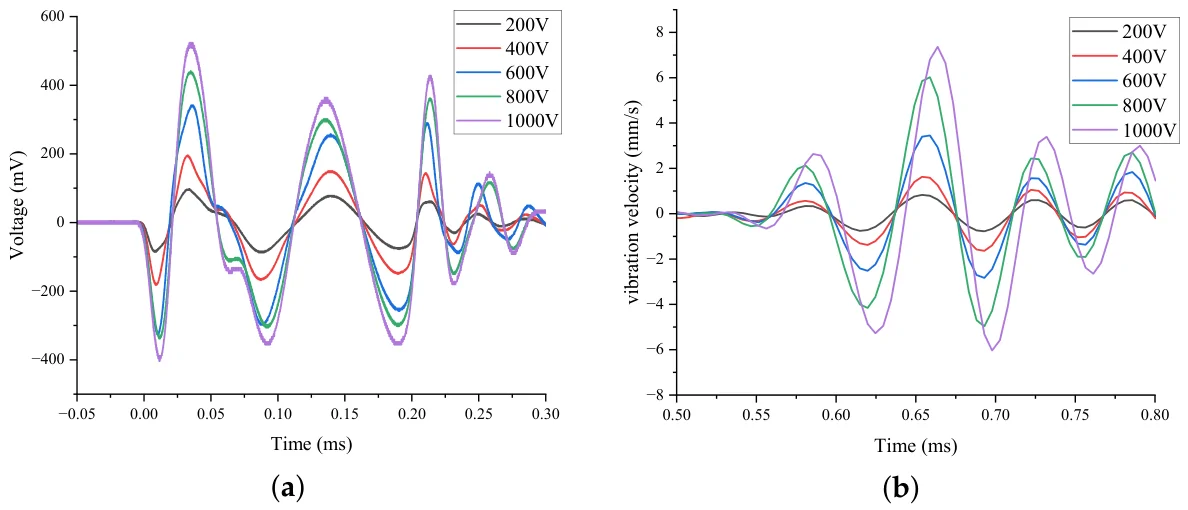
Waveform responses at different excitation voltages: (**a**) hydrophone-received voltage waveforms in the anechoic water tank; (**b**) first dominant vibration waveforms of the high-temperature vessel wall measured by laser vibrometry.

As shown in Figure 7, both the hydrophone-received voltage waveforms and the first dominant vessel-wall vibration waveforms exhibit consistent enhancement with increasing excitation voltage. This agreement supports the use of surface vibration velocity as a relative indicator of the transducer excitation response under otherwise unchanged conditions.

The two experiments measured different physical quantities under different propagation conditions: the anechoic-water-tank experiment recorded the hydrophone output voltage associated with the acoustic response, whereas the laser-vibrometry experiment measured the surface vibration velocity of the high-temperature vessel. Therefore, their absolute values and units cannot be compared directly. Nevertheless, when the transducer structure, mounting configuration, excitation waveform, and measurement position were kept unchanged and only the excitation voltage was varied, the waveform amplitudes obtained from both experiments increased consistently with increasing excitation voltage. This result indicates that the vibration response of the high-temperature vessel wall follows the same variation trend as the acoustic-radiation output of the piezoelectric transducer.

From a physical perspective, the piezoelectric transducer first converts electrical energy into mechanical vibration energy and subsequently radiates acoustic energy through coupling between its vibrating surface and the surrounding medium. When the dominant vibration mode and radiation load remain approximately unchanged, the acoustic-radiation output of the transducer is correlated with the vibration velocity of the vessel wall. Therefore, although the vibration velocity measured by laser vibrometry cannot be directly equated with the absolute acoustic-radiation energy of the transducer, it can effectively reflect relative variations in its mechanical excitation intensity and acoustic-radiation capability.

Because the correlation between vessel-wall vibration velocity and transducer output was established at room temperature, it was necessary to determine whether this relationship could be affected by the temperature-dependent acoustic properties of the silicone oil and the structural dynamics of the vessel. A control experiment was therefore conducted in which the vessel wall was excited directly by an electromagnetic hammer at the same impact position and with the same hammer supply current at each temperature. The hammer body remained outside the heated vessel at room temperature, thereby reducing the direct thermal influence on the actuator and providing nominally repeatable impact excitation. The measured response nevertheless included the combined effects of the temperature-dependent silicone-oil load and vessel dynamics.

**Figure 8:**
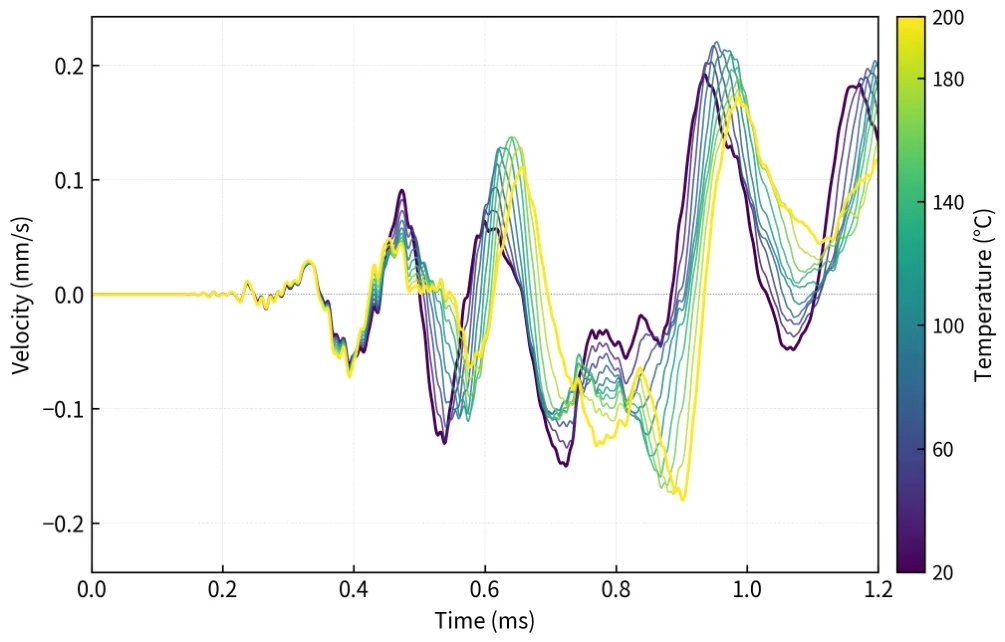
Vessel-wall vibration-velocity waveforms under electromagnetic-hammer excitation with constant supply current at different temperatures (20–200 °C; time window 0–1.2 ms).

Figure 8 presents the vessel-wall vibration-velocity waveforms measured from 20 to 200 °C. The waveforms were not identical at different temperatures: observable changes occurred in their phase, peak timing, and local shape, consistent with variations in the acoustic impedance of the silicone oil and the resonance characteristics of the vessel. However, these waveform changes did not result in a systematic decrease in the overall vibration-response strength. To avoid truncating the response as the peaks shifted with temperature, a common time window of 0–1.2 ms, encompassing the first dominant wall-vibration response at all temperatures, was used for the quantitative comparison. Within this window, the squared-velocity integral $J_{V}$ remained between approximately 98% and 102% of its value at 20 °C, while the maximum vibration velocity varied by approximately −7% to +15%. Neither indicator exhibited a monotonic temperature-dependent trend. By contrast, under constant-voltage transducer excitation, the measured $J_{V}$ decreased by approximately 81% over the same temperature range (Section 3.2). Therefore, although the temperature-dependent oil–vessel transfer path affects the detailed waveform and local peak response, it does not produce the pronounced systematic reduction observed under transducer excitation. This comparison indicates that the measured decrease is associated predominantly with the reduction in the transducer’s mechanical excitation output.

## 3. Results

### 3.1. Temperature Dependence of the Electrical Parameters of the Transducer

The resonant frequency ($f_{r}$) of a piezoelectric ceramic transducer is a key parameter characterizing its electromechanical coupling properties and vibration-response capability. It directly affects the optimal operating frequency range, energy-conversion efficiency, and transmitting and receiving performance of the transducer. Investigating the temperature dependence of $f_{r}$ not only helps reveal how temperature affects the material parameters and structural dynamic characteristics of the transducer, but also provides a basis for frequency design, performance evaluation, and stable operation under elevated-temperature conditions.

**Figure 9:**
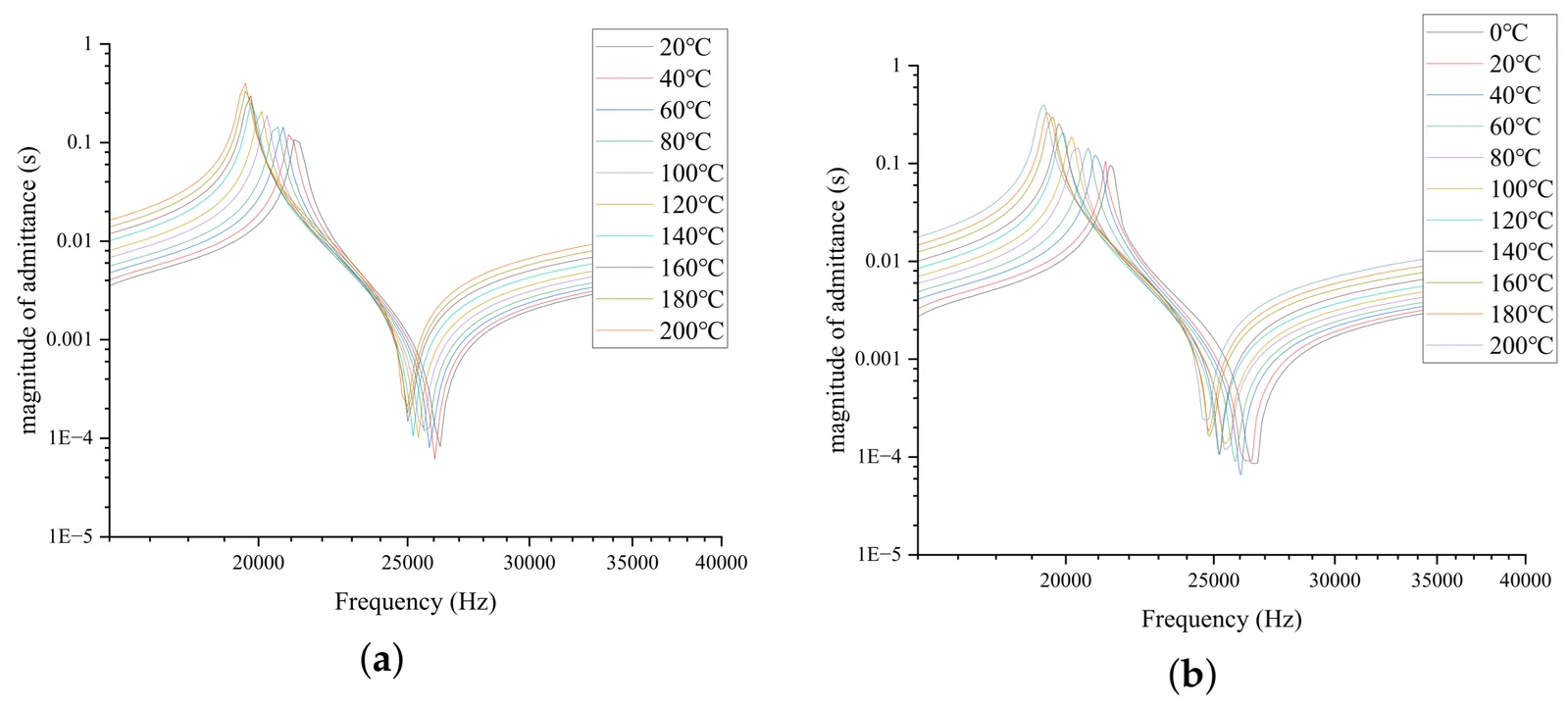
Admittance-magnitude curves near the dominant radial resonance at different temperatures: (**a**) impedance-experiment results; (**b**) impedance-simulation results.

Figure 9 shows the admittance-magnitude curves near the dominant radial resonance obtained from the impedance experiments and simulations at different temperatures. Both the experimental and simulated admittance curves exhibit a single dominant resonance peak within the analyzed frequency range. As the temperature increases, the resonance peak shifts progressively toward lower frequencies, while its magnitude increases markedly. The experimentally measured peak admittance increases from approximately 0.107 S at 20 °C to 0.400 S at 200 °C, corresponding to an increase of approximately 273%. The simulated peak admittance increases from approximately 0.107 S at 20 °C to 0.429 S at 200 °C, corresponding to an increase of approximately 301%. The close agreement between the experimental and simulated peak values at 20 °C, together with their consistent temperature-dependent evolution, indicates that the FE model reproduces the principal variation characteristics of the transducer admittance.

**Figure 10:**
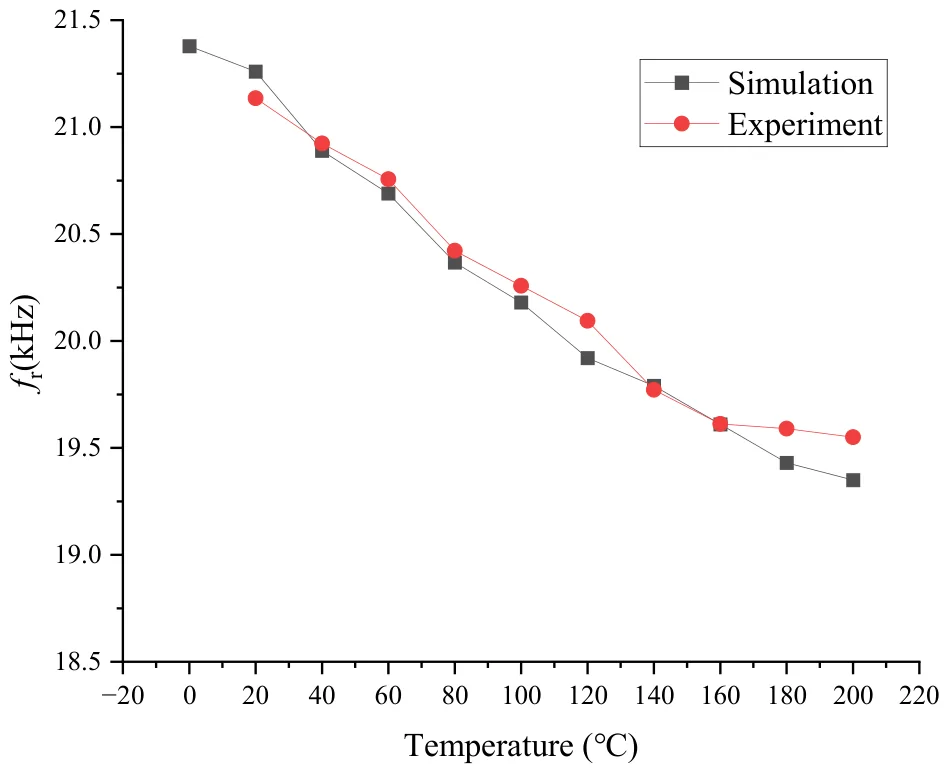
Temperature-dependent $f_{r}$ obtained from the impedance experiments and simulations.

Here, $f_{r}$ was identified as the frequency of the maximum admittance magnitude within the dominant radial-mode band. The corresponding values are presented in Figure 10.

As the temperature increases from 20 to 200 °C, the experimentally measured $f_{r}$ decreases from approximately 21.135 to 19.55 kHz, corresponding to a reduction of 7.5%. The simulated $f_{r}$ decreases from 21.379 kHz at 0 °C to 19.35 kHz at 200 °C, corresponding to a reduction of 9.5%. Overall, $f_{r}$ decreases approximately monotonically with increasing temperature, although slight variations in the local slope indicate that its temperature dependence is not strictly linear.

For the dominant radial mode, the resonant frequency is governed primarily by the effective modal stiffness, mass, geometric dimensions, and electromechanical coupling of the polarized ceramic. As the temperature increases while remaining below the Curie temperature, thermal activation enhances the reversible motion of ferroelectric domain walls and weakens defect-mediated domain-wall pinning. In particular, the increased activity of ${\mathrm{non-180}}^{\circ }$ domain walls contributes to an increase in the effective elastic compliance of PZT-5A, thereby reducing its dynamic stiffness. The resulting decrease in mechanical-wave velocity shifts the natural vibration frequency toward lower values. Meanwhile, the temperature-dependent evolution of the polarization state and piezoelectric coefficients further modifies the electromechanical coupling condition, which may account for the slight nonlinearity in the relationship between $f_{r}$ and temperature [15,22,32].

Therefore, the downward shift in $f_{r}$ can be regarded as a macroscopic manifestation of the combined evolution of the crystal lattice and ferroelectric-domain structure. The close agreement between the experimental and simulated trends over the entire temperature range indicates that the temperature-dependent constitutive parameters introduced into the finite-element model effectively capture the dominant material-property changes governing the resonant-frequency variation.

**Figure 11:**
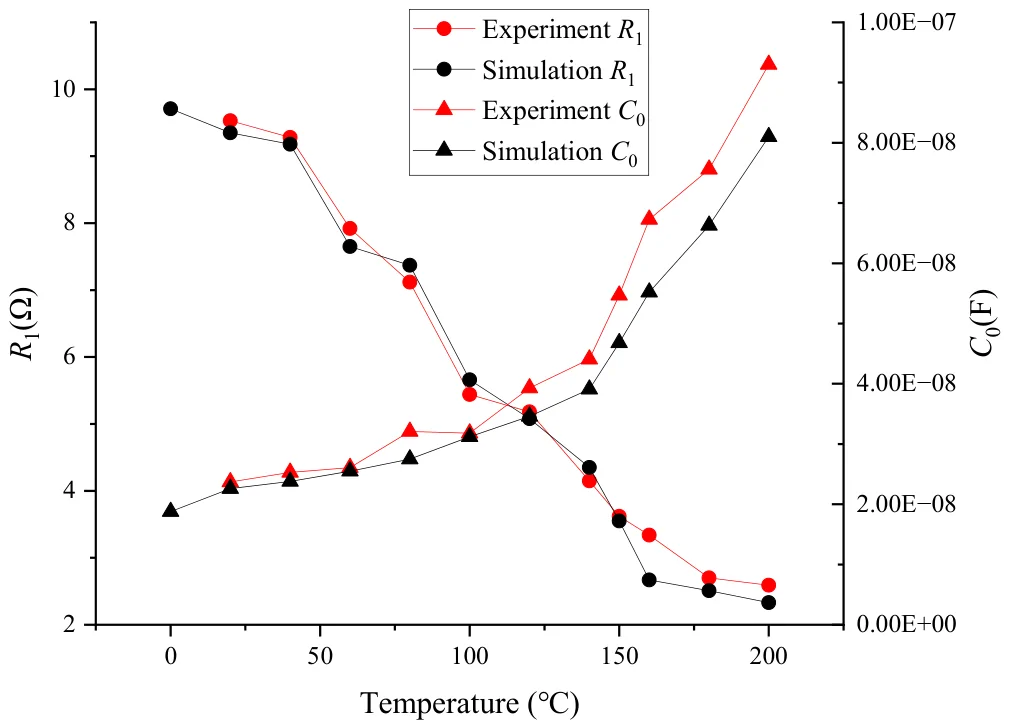
Curves of $R_{1}$ and $C_{0}$ versus temperature obtained from impedance experiments and simulations.

As shown in Figure 11, the equivalent-circuit parameters $R_{1}$ and $C_{0}$ obtained from the impedance experiments and simulations exhibit consistent temperature-dependent trends. Specifically, $R_{1}$ decreases monotonically as the temperature rises, whereas $C_{0}$ gradually increases with increasing temperature.

The experimental results show that $R_{1}$ decreases from 9.53 Ω at 20 °C to 2.59 Ω at 200 °C, while $C_{0}$ increases from 23.7 nF at 20 °C to 93.0 nF at 200 °C. The simulation results show that $R_{1}$ decreases from 9.71 Ω at 0 °C to 2.33 Ω at 200 °C, while $C_{0}$ increases from 18.8 nF at 0 °C to 81 nF at 200 °C. The simulated values of $R_{1}$ agree well with the experimental values. However, the simulated values of $C_{0}$ are generally lower than the experimental values, and the discrepancy between them increases with increasing temperature.

The fitted $R_{1}$ represents the resistance of the motional branch in the BVD equivalent circuit and should be interpreted as a macroscopic parameter of the complete electromechanical system. Its temperature dependence reflects the combined effects of elastic compliance, piezoelectric coefficients, dielectric and mechanical losses, domain-wall mobility, internal friction, and electromechanical coupling. Therefore, the decrease in the fitted $R_{1}$ indicates a reduction in the equivalent motional resistance of the fitted circuit, but it should not be interpreted as direct evidence of reduced intrinsic material loss or improved acoustic-output performance. This distinction is particularly important in the present study because the measured vibration-response and simulated pressure-response indicators decrease with temperature even though the fitted $R_{1}$ decreases. In contrast, the increase in $C_{0}$ is primarily associated with the temperature-dependent increase in the effective dielectric permittivity of PZT-5A [36].

However, the discrepancy between the simulated and experimentally measured $C_{0}$ values cannot be fully explained solely by the temperature-dependent dielectric parameters. Therefore, differences between the finite-element model and the actual experimental conditions must also be considered.

During actual heating, piezoelectric materials undergo thermal expansion as the temperature increases [37]. However, the geometric dimensions of the model cannot be directly modified during a parametric sweep based only on the global temperature parameter. Therefore, a separate geometric-expansion sensitivity simulation was conducted to investigate this possible influence. In this simulation, the total mass of the transducer and the other material parameters were held constant, while its dimensions and density were varied simultaneously. The simulation results are listed in Table 2.

As the transducer volume increases, corresponding to a decrease in density under the constant-mass condition, $R_{1}$ remains nearly unchanged, $C_{0}$ exhibits an increasing trend, and $f_{r}$ shows only slight fluctuations without a clear systematic variation. Geometric expansion occurs naturally during the impedance experiments; however, in the finite-element model, thermal expansion was not incorporated simultaneously with the temperature-parametric sweep of the material properties.

This modeling limitation may partly explain why the experimentally measured $C_{0}$ values in Figure 11 are higher than the simulated values and why the discrepancy increases with temperature, whereas no comparable deviation is observed for $R_{1}$ or $f_{r}$. Within this isolated geometric-expansion sensitivity analysis, the prescribed dimensional changes primarily affected the static capacitance, while its influence on the motional resistance and resonant frequency is relatively limited.

**Figure 12:**
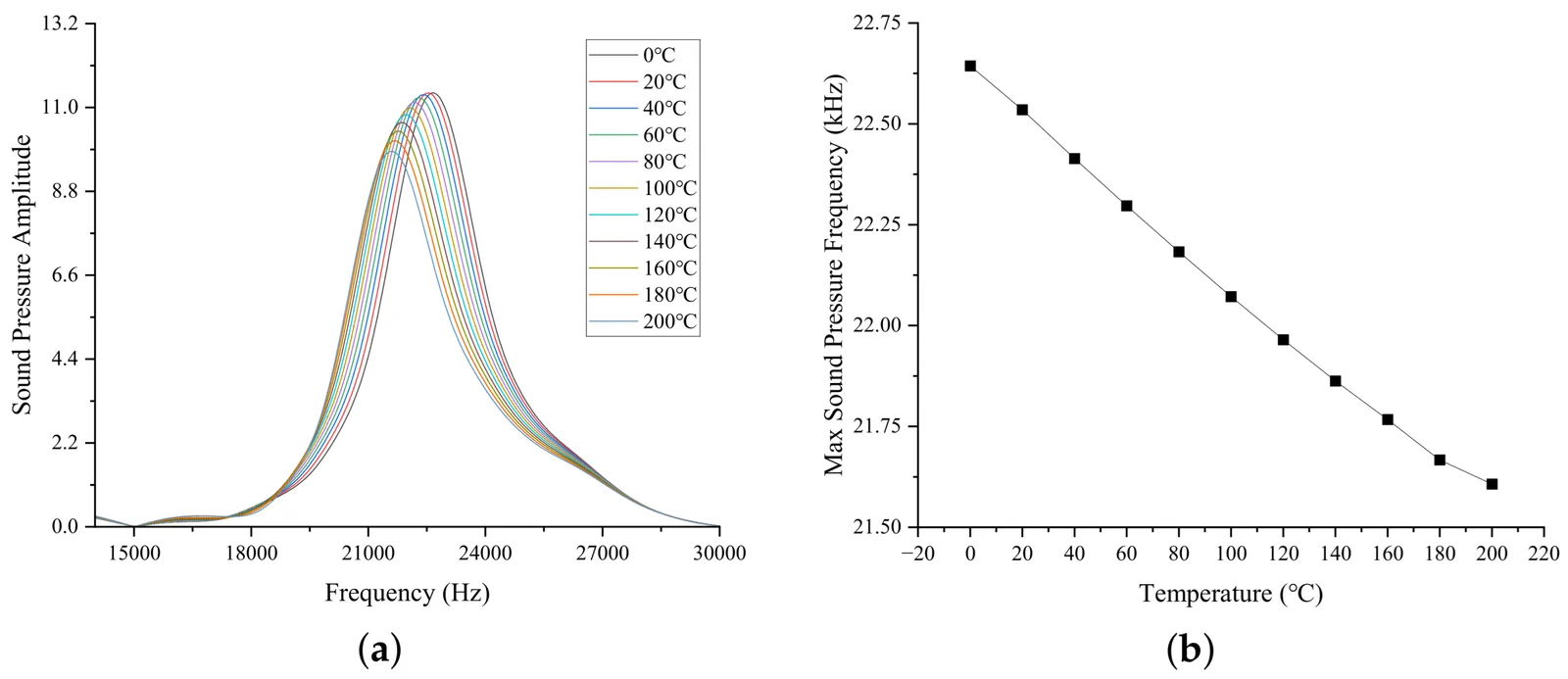
Temperature-dependent sound-pressure spectra and dominant-frequency variation: (**a**) sound-pressure spectra at different temperatures; (**b**) frequency corresponding to the maximum spectral sound pressure versus temperature.

Piezoelectric transducers generally operate under fluid-loaded conditions in practical applications, particularly in underwater acoustics and acoustic-logging systems. The surrounding fluid significantly affects the vibration characteristics, resonance response, and acoustic-radiation efficiency of the transducer. The transient probe responses were transformed to the frequency domain to obtain the spectra used in the following comparison. Figure 12a shows the sound-pressure magnitude spectra obtained from the sound-pressure probe located 1 m from the center of the transducer at different temperatures in the transient acoustic-radiation simulations. Figure 12b shows the temperature dependence of the frequency corresponding to the maximum spectral sound pressure.

The frequency corresponding to the maximum spectral sound pressure decreases monotonically from 22.644 kHz at 0 °C to 21.607 kHz at 200 °C. Although these absolute values are generally higher than the resonant frequencies obtained from the impedance experiments and impedance simulations, their temperature-dependent trend is consistent with those of the latter two. This result indicates that although the frequency corresponding to the maximum sound-pressure spectrum in water is not identical to the electrical resonant frequency determined from the impedance curve in air, both are governed by the temperature-dependent material parameters of the transducer.

### 3.2. Temperature Dependence of the Radiation Performance of the Transducer

The above analysis indicates that temperature variations not only alter the resonant frequency and equivalent-circuit parameters of the transducer but also significantly affect its electromechanical energy-conversion process. However, these electrical parameters primarily reflect the impedance characteristics and equivalent-circuit behavior of the transducer and are insufficient to directly characterize its mechanical vibration output and acoustic-radiation capability under actual operating conditions. The radiation performance of a piezoelectric transducer is ultimately manifested in its ability to generate mechanical vibration and radiate acoustic energy into the surrounding medium.

**Figure 13:**
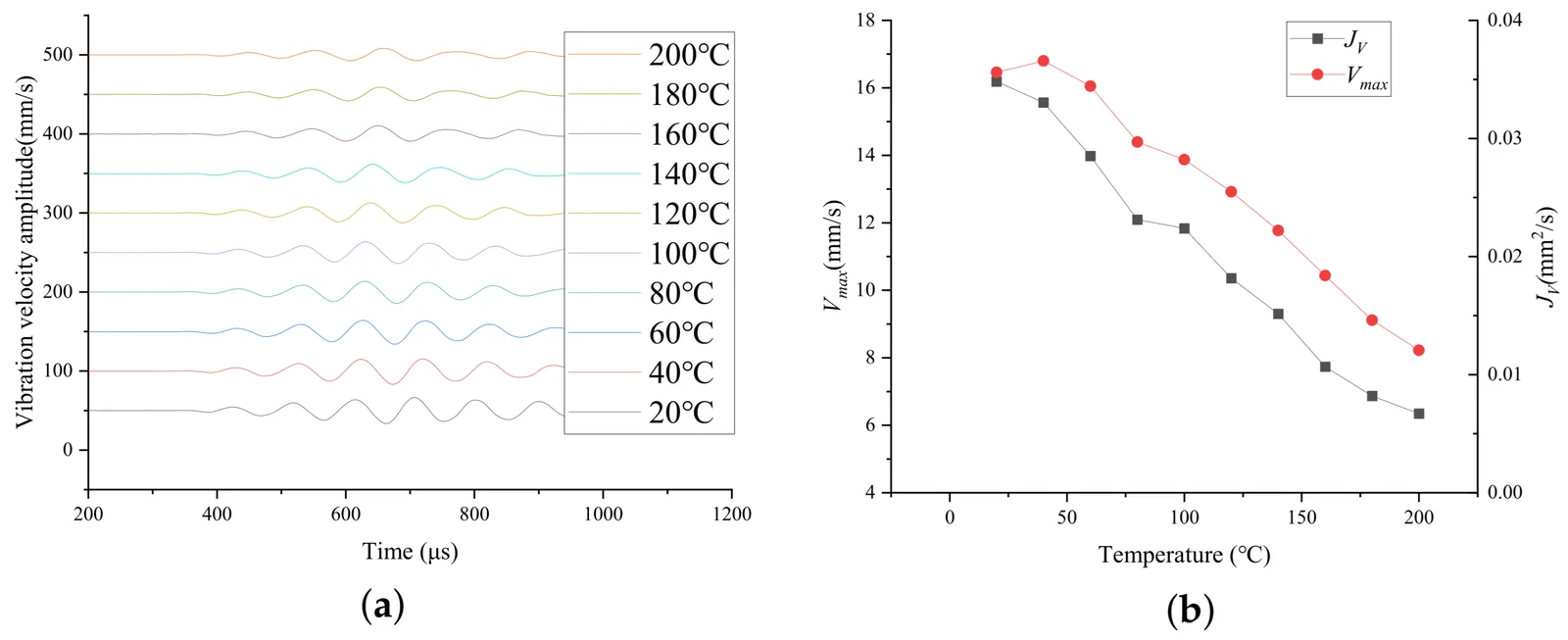
Temperature-dependent vessel-wall vibration responses measured by laser vibrometry: (**a**) first dominant vibration-velocity waveforms at different temperatures; (**b**) variations in $V_{\max}$ and $J_{V}$ with temperature.

Figure 13a shows the vibration-velocity curves of the first dominant vibration mode on the outer wall of the high-temperature vessel measured using the laser vibrometer over the temperature range of 20–200 °C. The amplitudes of the vibration responses generally decrease with increasing temperature, indicating that the mechanical excitation capability of the transducer is weakened under elevated-temperature conditions.

To quantitatively evaluate this variation, the maximum vibration velocity, $V_{\max}$, and the integral of the square of the vibration velocity, $J_{V}$, were calculated. Their temperature-dependent variations are shown in Figure 13b. Because the mechanical kinetic energy is proportional to the square of the vibration velocity, $J_{V}$ within a specified modal time window can be used as a relative indicator of the vibration-response strength when the measurement position, structural configuration, and excitation conditions remain unchanged:

(16) $$J_{V}=\int_{t_{1}}^{t_{2}}v^{2}\left(t\right)\;dt,$$

where $t_{1}$ and $t_{2}$ define the time window containing the same dominant vibration mode.

Compared with $V_{\max}$, which represents only the instantaneous peak response, $J_{V}$ accounts for both the vibration amplitude and duration and therefore provides a more comprehensive characterization of the overall mechanical vibration output of the dominant mode. As shown in Figure 13b, $V_{\max}$ initially increases and subsequently decreases over the temperature range of 20–200 °C, reaching a maximum of 16.80 mm/s at 40 °C. In contrast, $J_{V}$ decreases from $0.03483{\mathrm{mm}}^{2}$/s at 20 °C to $0.0067{\mathrm{mm}}^{2}$/s at 200 °C.

The local increase in $V_{\max}$ at 40 °C may result from its sensitivity to instantaneous waveform peaks, local modal-shape variations, and the measurement position. In contrast, $J_{V}$ represents the integral of the squared vibration velocity over the complete dominant-mode time window and is therefore less sensitive to a single local peak. Consequently, although the instantaneous maximum vibration velocity fluctuates at individual temperature points, the monotonic decrease in $J_{V}$ indicates that the overall mechanical excitation capability of the transducer continuously weakens with increasing temperature.

**Figure 14:**
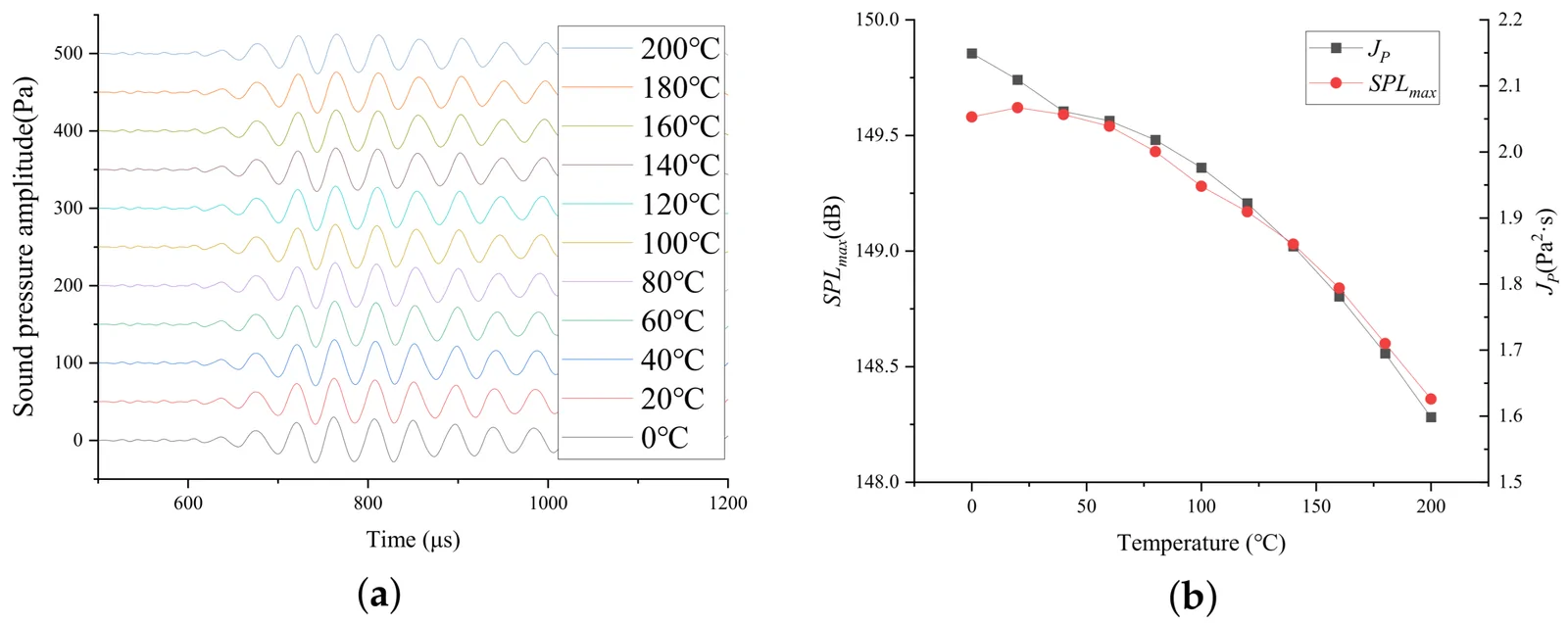
Temperature-dependent acoustic responses from the transient fluid-loaded simulations: (**a**) time-domain pressure curves at the probe located 1 m from the transducer; (**b**) variations in ${\mathrm{SPL}}_{\max}$ and $J_{P}$ with temperature.

Similarly, the maximum sound-pressure level (SPL), ${\mathrm{SPL}}_{\max}$, and the sound-pressure squared integral, $J_{P}$, were calculated from the transient acoustic-radiation simulation results. Figure 14a shows the time-domain sound-pressure responses at the probe located 1 m from the transducer center, and Figure 14b shows the corresponding values of ${\mathrm{SPL}}_{\max}$ and $J_{P}$. The maximum sound-pressure level was calculated using the underwater-acoustic reference pressure $p_{\mathrm{ref}}=1\;\mu \mathrm{Pa}$:

(17) $${\mathrm{SPL}}_{\max}=20\log_{10}\left(\frac{p_{\max}}{p_{\mathrm{ref}}}\right),$$

where $p_{\max}$ is the maximum absolute pressure within the selected response window.

The sound-pressure squared integral was calculated as

(18) $$J_{P}=\int_{t_{1}}^{t_{2}}p^{2}\left(t\right)\;dt,$$

where p(t) is the sound-pressure response at the probe position, and $t_{1}$ and $t_{2}$ define the selected acoustic-response time window. Similar to $J_{V}$, $J_{P}$ incorporates both the amplitude and duration of the acoustic-pressure response and can therefore be used as a relative indicator of the overall acoustic-radiation output.

As shown in Figure 14b, ${\mathrm{SPL}}_{\max}$ increases slightly at the beginning of the investigated temperature range, reaching a maximum of 149.62 dB at 20 °C, and then gradually decreases to 148.36 dB at 200 °C. In contrast, $J_{P}$ decreases continuously with increasing temperature, from 2.14926 Pa^2^·s at 0 °C to 1.59839 Pa^2^·s at 200 °C.

The slight variation in ${\mathrm{SPL}}_{\max}$ indicates that the instantaneous maximum sound-pressure response is sensitive to local waveform peaks and phase superposition. However, the continuous decrease in $J_{P}$ demonstrates that the overall acoustic-radiation output is progressively reduced as the temperature increases. Therefore, the laser-vibrometry measurements and transient acoustic-radiation simulations both indicate that the measured mechanical response and simulated acoustic response decrease overall with increasing temperature. The consistent reductions in $J_{V}$ and $J_{P}$ further indicate that the temperature-induced degradation extends from the mechanical vibration response to the acoustic-radiation output.

## 4. Discussion

The simulations and experiments show that temperature changes the electrical, mechanical, and acoustic responses of the tubular PZT-5A transducer in a coupled manner. The close agreement between the simulated and measured trends of $f_{r}$ and $R_{1}$ indicates that the implemented temperature-dependent constitutive functions capture the dominant change in the transducer’s electromechanical state. The remaining differences in absolute values are expected because the FE model assumes homogeneous material properties, uniform temperature, ideal electrodes, and simplified mechanical and electrical boundary conditions.

The downward shift in $f_{r}$ is consistent with the temperature-dependent increase in elastic compliance reported for PZT ceramics [15,16,18,19]. Below the Curie temperature, thermal activation can increase reversible domain-wall motion and weaken defect-mediated pinning, thereby increasing the extrinsic contribution to compliance and reducing the effective dynamic stiffness [24,27,28]. The resulting reduction in mechanical-wave velocity shifts the dominant radial resonance toward lower frequencies. Changes in polarization state and piezoelectric coefficients can further modify the electromechanical coupling and contribute to the nonlinearity of the frequency–temperature relationship. Studies in which domain configurations were deliberately altered by alternating-current poling provide additional evidence that domain structure can strongly modify dielectric and piezoelectric properties, although those studies do not directly represent a thermal process [30,31].

The increase in $C_{0}$ is primarily associated with the increase in effective dielectric permittivity, including both intrinsic lattice-polarization and extrinsic domain-wall contributions [17,36]. The measured $C_{0}$ values were higher than the simulated values, and the discrepancy increased with temperature. In the isolated geometric-expansion sensitivity analysis, prescribed dimensional changes increased $C_{0}$ while producing only small changes in $R_{1}$ and $f_{r}$. This result suggests that omission of thermal deformation may contribute to the capacitance discrepancy, but it should not be interpreted as a complete quantitative model of thermal expansion. Electrode layers, lead wires, temperature gradients, manufacturing tolerances, and temperature-dependent dielectric loss may also contribute.

The fitted $R_{1}$ should be interpreted as a macroscopic BVD parameter of the complete electromechanical system rather than as a direct measurement of a single intrinsic loss mechanism. Dielectric loss, elastic compliance, piezoelectric coefficients, domain-wall mobility, internal friction, and coupling conditions can all change the fitted motional branch. Consequently, a decrease in $R_{1}$ does not necessarily imply improved acoustic output. This point is important because the vibration and pressure indicators decreased overall even while the fitted motional resistance became smaller.

The laser-vibrometry measurements and transient fluid-loaded simulations extend the analysis beyond the electrical response. The local fluctuation in $V_{\max}$ illustrates the sensitivity of a point measurement to instantaneous peaks, mode-shape changes, and the selected observation location. By integrating the squared response over a common time window, $J_{V}$ and $J_{P}$ account for both amplitude and duration and are less sensitive to a single peak. Their consistent decrease indicates an overall weakening of the measured vessel-wall vibration response and simulated pressure response with temperature. Nevertheless, neither quantity is an absolute energy measurement: $J_{V}$ is derived from a single point on the high-temperature vessel, whereas $J_{P}$ is derived from a fixed pressure probe and does not include the full radiation area, medium impedance, or three-dimensional directivity.

The electromagnetic-hammer control experiment described in Section 2.3 indicates that the temperature-dependent silicone-oil load and vessel dynamics alter the waveform but do not introduce a systematic reduction in the integrated wall-vibration response. Therefore, the much larger monotonic decrease observed under transducer excitation cannot be explained primarily by the temperature-dependent oil–vessel transfer path and is interpreted mainly as a reduction in the transducer’s mechanical excitation output. Nevertheless, the vibration velocity measured at a single point on the vessel wall represents only the relative response of the coupled transducer–oil–vessel system and should not be regarded as an absolute measure of radiated acoustic energy.

For acoustic-logging applications, the coupled variations in $f_{r}$, $C_{0}$, $R_{1}$, vibration response, and radiated pressure imply that excitation and impedance-matching conditions optimized at room temperature can become suboptimal downhole. Temperature-dependent frequency tracking, adaptive matching, and excitation-voltage compensation are therefore relevant engineering strategies [13]. The present model provides a basis for such compensation, but further work should couple thermal deformation and thermally induced stress with temperature-dependent dielectric and mechanical losses. Complete temperature-dependent data were unavailable for $d_{15}$, $s_{13}^{E}$, $s_{33}^{E}$, and $s_{55}^{E}$, which were therefore held at their room-temperature values. In addition, the model represents domain evolution through macroscopic constitutive functions rather than explicitly resolving the domain structure. Future studies should incorporate more complete material functions, spatial temperature gradients, and direct microstructural measurements or phase-field modeling.

## 5. Conclusions

A temperature-dependent FE model was established for a tubular PZT-5A transducer and assessed using impedance measurements and laser-vibrometry experiments. The principal conclusions are as follows.

1. The electrical response showed pronounced temperature dependence. Over 0–200 °C, the simulated $C_{0}$ increased by 330.9%, $R_{1}$ decreased by 76.1%, and $f_{r}$ decreased by 9.5%. Over 20–200 °C, the measured $C_{0}$ increased from 23.7 to 93.0 nF, $R_{1}$ decreased from 9.53 to 2.59 Ω, and $f_{r}$ decreased from 21.135 to 19.55 kHz. The consistent trends in $f_{r}$ and $R_{1}$ support the ability of the model to reproduce the dominant temperature-dependent electromechanical behavior.
2. The measured $C_{0}$ was systematically higher than the simulated value. In the isolated geometric-expansion sensitivity analysis, the prescribed dimensional increase raised $C_{0}$ from 18.8 to 23.7 nF while producing only small changes in $R_{1}$ and $f_{r}$. Omitted thermal deformation may therefore partly explain the capacitance discrepancy, although additional experimental and modeling factors are also likely involved.
3. The mechanical and acoustic response indicators decreased overall with temperature. $J_{V}$ decreased from $0.03483{\mathrm{mm}}^{2}$/s at 20 °C to $0.0067{\mathrm{mm}}^{2}$/s at 200 °C, while $J_{P}$ decreased from 2.14926 to 1.59839 Pa^2^·s over 0–200 °C. The frequency corresponding to the maximum sound-pressure spectrum decreased from 22.644 to 21.607 kHz, consistent with the downward shift of the impedance-derived resonance. Because both squared-integral quantities were obtained at fixed observation positions, they are relative response indicators rather than absolute energy values.
4. The principal scientific contribution of this study is the establishment of an experimentally constrained cross-domain relationship among the temperature-dependent constitutive properties of PZT-5A, the electrical equivalent-circuit parameters, the mechanical vibration response, and the fluid-loaded acoustic response of a tubular transducer. The decrease in the fitted motional resistance accompanied by the overall reductions in $J_{V}$ and $J_{P}$ demonstrates that temperature-dependent electrical parameters alone are insufficient to represent the evolution of mechanical and acoustic outputs. The electromagnetic-hammer control experiment further indicates that the systematic reduction in the measured vessel-wall vibration response is dominated by changes in the transducer response rather than by the temperature-dependent silicone-oil–vessel system. These findings provide a physical basis for the performance evaluation, frequency tracking, impedance matching, and temperature compensation of tubular piezoelectric transducers used in acoustic-logging applications.

Future work should include fully coupled thermal deformation and internal stress, temperature-dependent dielectric and mechanical losses, and more complete constitutive data to improve quantitative agreement between simulations and experiments.

## Figures and Tables

**Table 1:** Temperature-dependent material parameters of PZT-5A used in the simulation.

Temperature, Θ (°C)	$\varepsilon_{11}^{T}/\varepsilon_{0}$	$\varepsilon_{33}^{T}/\varepsilon_{0}$	$d_{31}$ (pC/N)	$d_{33}$ (pC/N)	$s_{11}^{E}$ ($10^{-12}$ $m^{2}$/N)	$s_{12}^{E}$ ($10^{-12}$ $m^{2}$/N)
0	1524.3	1624.3	−147.9	353.6	15.4023	−5.0653
20	1623.7	1723.7	−169.9	362.8	15.4833	−5.0161
40	1732.6	1832.6	−189.5	371.6	15.5414	−4.9424
60	1851.2	1951.2	−206.7	380.1	15.5766	−4.8439
80	1979.3	2079.3	−221.6	388.3	15.5887	−4.7209
100	2117.0	2217.0	−234.0	396.1	15.5780	−4.5732
120	2264.3	2364.3	−244.1	403.7	15.5442	−4.4008
140	2421.2	2521.2	−251.8	410.9	15.4875	−4.2038
160	2587.7	2687.7	−257.1	417.8	15.4079	−3.9822
180	2763.8	2863.8	−260.1	424.3	15.3053	−3.7359
200	2949.5	3049.5	−260.7	430.0	15.1798	−3.4650

**Table 2:** Impedance-simulation sensitivity results for prescribed geometric expansion.

Density	Outer	Inner	Height	$R_{1}$	$C_{0}$	$f_{r}$
(${\mathrm{kg/m}}^{3}$)	Diameter (mm)	Diameter (mm)	(mm)	(Ω)	(F)	(kHz)
7750	70.00	60.00	76.00	9.71	$1.88\times 10^{-8}$	21.37
7598	70.46	60.40	76.50	9.71	$2.13\times 10^{-8}$	21.42
7410	71.05	60.90	77.14	9.71	$2.37\times 10^{-8}$	21.39

## Data Availability

The data presented in this study are available from the corresponding author upon reasonable request.

## Abbreviations

BVD	Butterworth–Van Dyke
FE	Finite element
PML	Perfectly matched layer
PZT	Lead zirconate titanate
SPL	Sound-pressure level
